# Sensory Determination of Peach and Nectarine Germplasms with Instrumental Analysis

**DOI:** 10.3390/foods12244444

**Published:** 2023-12-11

**Authors:** Meng Sun, Julin Ma, Zhixiang Cai, Juan Yan, Ruijuan Ma, Mingliang Yu, Yinfeng Xie, Zhijun Shen

**Affiliations:** 1 Institute of Pomology, Jiangsu Academy of Agricultural Sciences, No. 50 Zhongling Street, Nanjing 210014, China; sm183495665@163.com (M.S.);; 2 Jiangsu Key Laboratory for Horticultural Crop Genetic Improvement, Jiangsu Academy of Agricultural Sciences, No. 50 Zhongling Street, Nanjing 210014, China; 3 Co-Innovation Center for Sustainable Forestry in Southern China, College of Biology and the Environment, Nanjing Forestry University, Nanjing 210037, China

**Keywords:** *Prunus perica* (L.) Batsch, germplasm, palate, flavour, sugars, acids

## Abstract

The flavour and mouthfeel of peaches are crucial qualities of peach germplasm resources that significantly influence consumer preferences. In this study, we utilized 212 peach germplasm resources from the Nanjing Peach Resource Repository, National Fruit Germplasm facility, Jiangsu Academy of Agricultural Sciences as materials for sensory analysis, electronic nose analysis, and composition analysis via high-performance liquid chromatography (HPLC). In the sensory analysis, we divided 212 peach germplasms into three clusters based on hierarchical cluster analysis (d = 5). No.27, No.151, and No.46 emerged as the most representative of these clusters. The electronic nose was used to conduct an evaluation of the aroma profiles of the 212 peach germplasms, revealing that the primary distinguishing factors of peach aroma can be attributed to three sensors: W1S (methane), W1W (terpenes and organosulfur compounds), and W5S (hydrocarbons and aromatic compounds). The primary differences in the aromatic substances were characterized by sensors W2W (aromatic compounds, sulphur, and chlorine compounds) and W1C (aromatic benzene). The HPLC analysis indicated that the persistence of peach sensory characteristics was positively correlated with acids and sourness and negatively correlated with sweetness and the ratio of sugar to acids. The overall impression of the 212 peach germplasms revealed a negative correlation with acids, while a positive correlation was observed between the overall impression and the ratio of sugar to acids. Therefore, this study substantially contributes to the preliminary screening of the analysed specific characteristics of peach germplasms such as No.27, No.46, No.151, and No.211. These selections may provide valuable information for the potential creation of superior germplasm resources.

## 1. Introduction

Peaches (*Prunus persica* (L.) Batsch) are the most important fruits in the world. Peaches are native to China and widely planted over 10,000 hectares (15.02 million tons, FAOSTAT, 2020), accounting for over 60% of the global total of peaches and nectarines. The evaluation of peach germplasm resources is important for their protection and utilization and the full exploitation of their potential economic, social, and ecological value in China [1]. The evaluation indexes used in this regard include morphological and biological characteristics, quality characteristics, stress resistances, and pest resistance. Recent studies have mainly focused on morphological and quality characteristics [2].

Peach fruit quality is composed of fruit appearance; internal, nutritional, and flavour quality; storage qualities; and stress tolerance [3]. Sensory evaluation, electronic nose analysis, electronic tongue analysis, and GC-MS are used to assess fruit flavour and palate as well as in comprehensive evaluation methods. Sensory evaluation involves the comprehensive and objective evaluation of the colour, aroma, taste, and appearance of food using human sensory organs (eyes, nose, mouth, teeth, hands, etc.), providing real data for mathematical and statistical analyses [4]. Previous research has been conducted on fruit wine and strawberry jam [5,6]. Sensory quality analysis plays a crucial role in the development of new food varieties, market prediction, setting product standards, and determining product shelf life [7]. Liu et al. [8] used fuzzy comprehensive judgment and the percentage total score method to objectively evaluate the colour, taste, and flavour of canned yellow peaches. However, there is limited research on the sensory analysis of fresh peach germplasm resources.

An electronic nose is a sensitive device used to detect a wide range of volatile compounds through the overall response of a specific sensor and a pattern recognition system [9]. Its advantages are its simplicity, rapidity, and facilitation of non-destructive sample analysis and automatic data collection. It is widely used in food testing, environmental monitoring, air-quality monitoring, and healthcare [10]. In the context of fruits, it can be used for variety identification, harvest and shelf-life judgement, disease identification, and the determination of freshness, ripeness, and decay [11]. An electronic nose not only enables the comparison and analysis of the odour information from different samples but also facilitates the establishment of a database by collecting the fingerprints of standard samples. This database can be used for the qualitative and quantitative analysis of the unknown components in samples [12]. 

This study combines sensory evaluation and electronic nose analysis with HPLC to explore the differences in aroma and palate among peach fruits with different flesh types and colours in 212 peach germplasms. The findings contribute to the selection, breeding, and utilization of excellent peach germplasms and improve the evaluation methods for peach germplasm resources, meeting the new demand pertaining to production and consumption. 

## 2. Method and Materials

### 2.1. Plant Materials

The 212 varieties of peaches/nectarines (Appendix A) analysed were harvested at the National Peach Germplasm Repository (Nanjing, China, 32°20′ N, 118°52′ E, located 11 m above sea level) from May to July 2022, with each variety represented by two trees. The test varieties were 5-year-old mature trees with a Y shape and a row spacing of 3 to 5 m, and conventional cultivation measures were employed. Samples (50 peaches/nectarines) of each variety were randomly collected upon reaching maturity (defined as the disappearance of green coloration on the bottom of the fruit) from the two trees [13,14]. Samples were harvested before 11:00 a.m. A total of 30 fruits of each variety were immediately stored in a freezer (−80 °C) for E-nose and HPLC measurement. A total of 20 fruits of each variety were taken into the sensory room for evaluation the following afternoon.

### 2.2. Sensory Analysis

The quantitative descriptive analysis method was used to train the sensory panel and carry out the sensory analysis according to the Sensory Analysis–Methodology Paired comparison test (ISO, 5495:2005) [15]. Different samples of peaches and nectarines were used to allow the panel members to recognize their qualitive characteristics. Based on their availability, health, and general food habits, ten candidates (postgraduate students and researchers from JAAS, comprising five females and five males) were pre-screened. They volunteered for the project. All subjects gave their informed consent for inclusion before they participated in the study. In this study, materials were taken from the National Peach Germplasm Repository, Nanjing, China. These materials are safe for sensory research. All the experimental procedures involving volunteers were conducted in accordance with the Declaration of Helsinki. Also, the volunteers had the ability to discriminate between products according to basic taste thresholds and to describe their perceptions. Panel descriptions and definitions for peach and nectarine attributes (flavour, palate, persistence, and overall impression) were developed through brainstorming and round-table consensus. 

The next step was to develop the score sheet for the different attributes for analysis. Flavour was evaluated according to being fruity, floral, earthy, woody, caramel, nutty and herbaceous, or vegetative. Palate (aroma, sweetness, sourness, and bitterness and astringency), persistence, and over impression were evaluated by assigning categorical scores from 0 to 10 (0, no sensation; 0.1–1.9, absence of sensation; 2.0–4.9, weak; 5.0–7.9, moderate; 8.0–10, intense) [16]. Each of them independently estimated 20 fruits of typical size and appearance and with similar ripeness per germplasm. Then, each panellist randomly chose two fruits from each variety for palate evaluation. The evaluated samples were not named until the end of the evaluation. The evaluations were judged at 10 a.m in individual booths under white light. 

### 2.3. E-Nose Measurement for Fruit Aroma

In total, 15 fruits were randomly divided into 3 blocks for E-nose measurement and analysed in triplicate. Measurements were performed using a portable, commercially available E-nose (PEN3.5, Airsense Analytics GmbH, Schwerin, Germany). The sensor array of the PEN3.5 has ten metal oxide semiconductor-type chemical sensors (Table 1) capable of operating at high temperatures to permit the classification and identification of different volatile species. When the sensors were exposed to volatiles, the changes in the conductivity (G)-to-initial-conductivity (G0) ratio (G/G0, relative conductivity or response value) depended on G conductivity. The concentration of volatiles led to the deviation of G/G0 (greater than or less than 1). In this study, 10 g of flesh from each sample was placed into a 300 mL beaker covered with sealing film. The beaker was kept at 25 °C for 30 min for E-nose evaluation. The aroma measurement method used followed that reported by Yan et al. [17], with some modifications. We inserted the E-nose sampling needle into the sealing film and extracted the gas in the beaker for detection. The volatile gas was pumped over the sensors of the E-nose at a flow rate of 400 mL/min. The E-nose analyses were recorded over a range from 0 to 60 s. One to three stable signals (response values) were observed to be in the middle period of the data stabilization time [18]. After each analysis, the sampling chamber was washed with an air-dried flow for 60 s. 

### 2.4. HPLC Measurement for Sugars and Acids

A total of 15 fruits were randomly divided into 3 groups for HPLC measurement and analysed in triplicate. The method of HPLC measurement for sugars and acids followed was modified by Shen et al. [19]. Using 5 mL of extracting solution (ethanol: 0.2% metaphoric acid *v*/*v*), 0.5 g of the flesh was ground and extracted using ultrasound waves for 1 h. The solution was centrifuged for 5 min at 10,000 rpm and 4 °C. The 0.8 mL supernatant was dried using a concentrator (Eppendorf, Concentrator plus, Sigma Aldrich, Saint Louis, MO, USA, 230 V/50–60 Hz) for 2 h. A total of 1.6 mL of ultrapure water was added to the concentrate to obtain sugar/organic acid solution. The samples were filtered through a 0.22 μm nylon syringe filter into an HPLC glass vial and capped tightly. Then, 0.5 μL portions of the samples were injected into an HPLC system with a 10 μm, 250 × 6 mm CARBOSep CHO-620 CA column (Transgenomic, Omaha, NE, USA) to detect soluble sugars at 80 °C using a differential refractive index detector. For the mobile phase, we used ultrapure water added at 0.5 mL min^−1^.

A total of 0.5 μL of the samples was injected into an HPLC system with a 5 μm, 250 × 4.6 mm ZORBAX Eclipse XDB-C18 column (Agilent, Santa Clara, CA, USA) to analyse organic acids at 2 °C using a VWD UV detector (λ = 214 nm). The mobile phase was 0.02 mol/L of KH_2_PO_4_ (pH 2.7) for 0.5 mL min^−1^. Data were analysed using the Chemstation (Agilent) chromatography data system. KH2PO4 was used as a standard for each batch of samples. A total of 0.02 mol/L of KH_2_PO_4_ (pH 2.7) (3.333, 1.667, 0.333, 0.167, and 0.033 g/L) was used to establish a standard curve for reporting organic acids. Soluble sugars and organic acids were identified by their retention times, and their concentrations were calculated in parallel to calibrate the internal ultrapure water and 0.02 mol/L of KH_2_PO_4_ (pH 2.7) standard curves.

### 2.5. Statistical Analysis

Sensory and HPLC data were tested in terms of their average characteristics at maturity using IBM SPSS Statistics 22 (IBM, Armonk, NY, USA). Pearson correlation test (*p* < 0.05) was conducted to analyse the relationship between sensory indexes and sugars and acids. Principal component analysis (PCA) and hierarchical cluster analysis were performed using OriginPro 2021 (OriginLab Corporation, Northampton, MA, USA). Data related to E-nose were processed using the WinMuster program in PEN3.5 (Airsense Analytics GmbH, Schwerin, Germany) and OriginPro 2021. The main sensors used for main peach smell detection were analysed via loading analysis (LOA) using WinMuster software (Version 1.6.2.15–2011, Airsense Analytics, Schwerin, Germany). Principal component analysis (PCA) and hierarchical cluster analysis were performed using OriginPro 2021 based on main and aromatic substance sensors.

## 3. Results

### 3.1. Sensory Analysis

**Figure 1:**
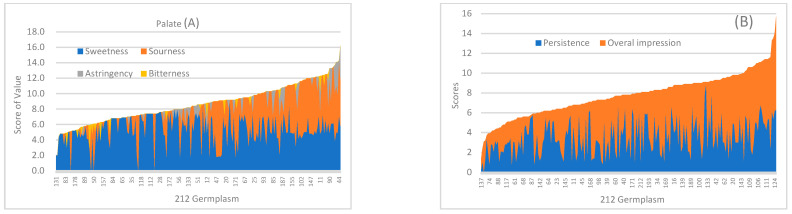
(**A**). Palate scores of 212 peach germplasm resources: blue area corresponds to sweetness scores; orange area corresponds to sourness; grey area corresponds to astringency; and yellow area corresponds to bitterness. (**B**). Persistence and overall impression scores of 212 peach germplasm resources: blue area corresponds to persistence, and orange area corresponds to overall impression.

The sensory evaluation results for the 212 peach germplasms are shown in Appendix A, including the scores for sweetness (0–9.2), sourness (0–7.5), astringency (0–6.3), and bitterness (0–3.1). In Figure 1A, sweetness has a larger area than sourness, astringency, and bitterness. Five germplasms exhibited intense sweetness (8–10 points); five germplasms elicited no sensation (0); and nine germplasms provoked a near absence of sensation (0.1–1.9). The most prominent sweetness was that of No.134. Regarding sourness, 79 resources elicited no sensation of sourness, 33 scored between 0 and 2.4, and 101 scored between 2.5 and 7.5. Only a small percentage of germplasms elicited astringent or bitter sensations. Astringency was weak in 17 germplasms and moderate in No.200. Three germplasms (No.2, No.11, and No.37) had weak bitterness.

Persistence was described as the duration of flavours and aromas for a peach, ranging from none to long. As indicated in Figure 1B, No.164 showed no persistence, 39 germplasms had a short finish (0.1–1.9), and 112 peach fruits exhibited short to medium persistence (2.0–4.9), constituting the largest group in the study. No.38, No.47, and No.119 had long finishes (8.0–10.0). Overall impression encompassed final thoughts with a rating (score out of 10) and freestyle impressions, aiding in distinguishing the participants’ favourite peaches. Germplasms were evaluated as excellent (8.0–10.0), good (5.0–7.9), medium (2.0–4.9), and poor (0.1–1.9), with 40 poor, 88 medium, 77 good, and 7 excellent germplasms. No.76 had a poor overall impression, while the highest scores for excellent impressions were those for No.209 and No.134. 

‘The Davis Wine Aroma Wheel’ is an ideal tool for identifying and visualising the various tastes, smells, and aromatic qualities in most wines, regardless of grape variety. According to this wheel, the 212 peach germplasms contain special flavours such as fruity (passion fruit, lemon, citrus, apple, green apple, honeydew, mango, apricot, juicy honey peach, and yellow peach), floral (gardenia, jasmine, rose, and violet), herbaceous or vegetative (herb, grass, celery, carrot, and lotus seed), caramel (candy, popcorn, molasses, and honey), woody (smoky and rubber), nutty (peanut, cereal, and red bean), animal (butter/milk), and pungent (metal) as well as faults like being spoiled and exuding off-odours.

Table 2 indicates that 137 germplasms possessed one or more special flavours, while 75 germplasms lacked any special flavour. Notably, No.15 and No.196 each had three distinct flavours (lemon, gardenia, and smoky for No.15; mango, violet, and butter/milk for No.196). A total of 29 germplasms had a violet aroma, and 31 germplasms had a butter/milk aroma. Certain aromas were unique to one germplasm, such as apricot (No.207), herb (No.173), celery (No.137), lotus seed (No.168), carrot (No.118), and rubber (No.111). Seven germplasms exhibited faults, including being spoiled (No.38, No.50, and No.76) and exuding an off-odour (No.11, No.123, No.160, and No.199).

**Figure 2:**
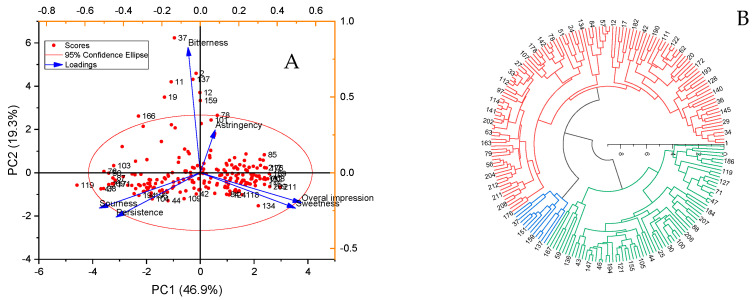
(**A**). Principal component analysis (PCA) of sensory evaluations for 212 peach germplasm resources: red dots, 212 peach germplasms; blue arrow lines, loadings. (**B**). Hierarchical cluster analysis of sensory analysis for 212 peach germplasm resources: red, Class I germplasms; green, Class II germplasms; blue, Class III germplasms.

Principal component analysis (PCA) revealed the influence of each sensory descriptor on the evaluation of peach germplasm resource (Figure 2A). The first principal component (PC1) accounted for 46.1% of the variance, with sweetness and overall impression contributing significantly. Sourness and persistence also had negative loadings for PC1. The variance contribution of the second principal component (PC2) was 19.2%, with astringency and bitterness serving as major contributors. Thus, PC1 reflected an enjoyable taste, while PC2 was associated with negative feedback. No.211 was characterized as sweet with a great impression, while No.101 was perceived as astringent and bitter with an unpleasant impression. No.119 had light sweetness, strong sourness, and a long finish, resulting in a harsh impression. Additionally, sweetness correlated closely with overall impression, as did sourness with persistence. Cluster analysis categorized the 212 germplasms into three groups (Figure 2B), with 127 in cluster 1, 13 in cluster 2, and 72 in cluster 3. No.27, No.151, and No.46 were the most representative of their respective clusters.

### 3.2. Instrumental Analysis

#### Response to Different Sensors Evaluated via E-Nose

**Figure 3:**
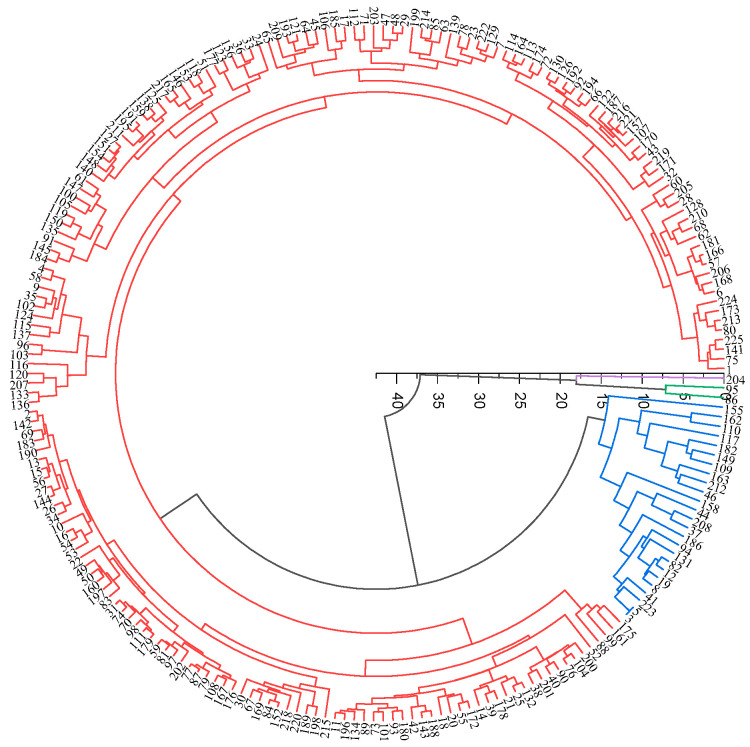
Hierarchical cluster analysis of E-nose performance for 212 peach germplasm resources: red, Class I germplasms; blue, Class II germplasms; green, Class III germplasms.

Based on the response values of 10 sensors, 212 peach germplasm samples were divided into four categories (d = 15) (Figure 3): 186 were assigned to Class I, 23 were assigned to Class II, 2 were assigned to Class III, and 1 was assigned to Class IV. Consequently, No.82, No.91, and No.193 were deemed notable for their distinction from Class I and II.

**Figure 4:**
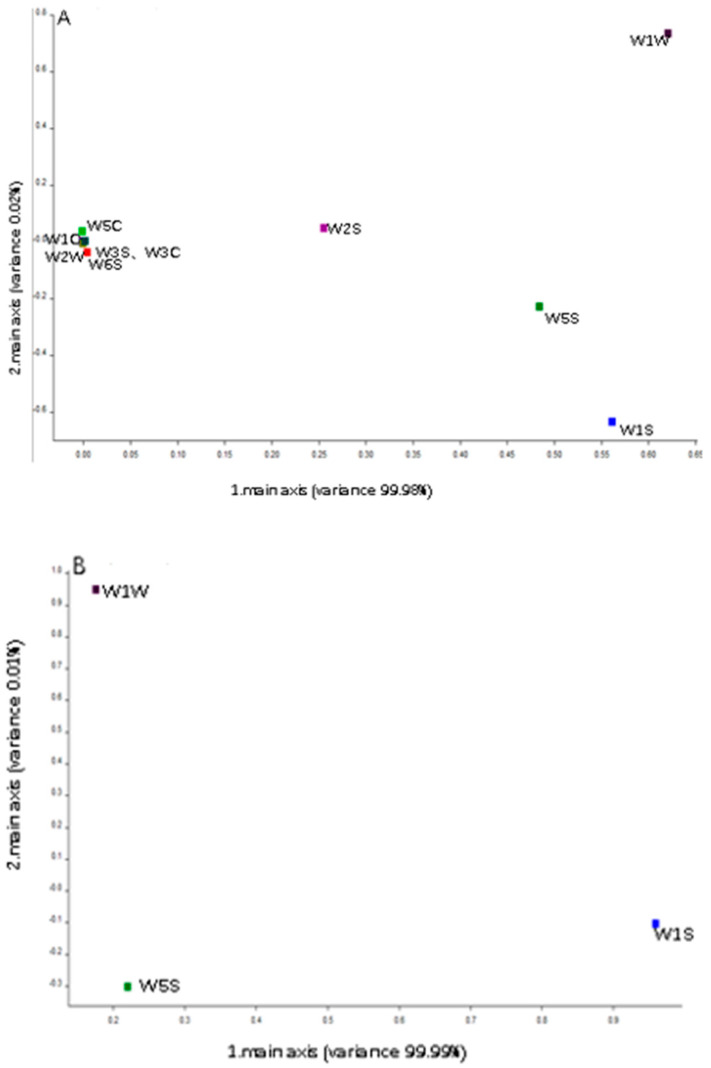
Aroma loading analysis conducted using E-nose in relation to 212 peach germplasm resources ((**A**): ten sensors; (**B**): three sensors).

In Figure 4A, the W1W sensor had an influence on both the first and second principal components. The sensors W1S and W5S contributed to the first principal component. To validate the significance of sensors W1W, W1S and W5S in differentiating the peach fruit aromas, the three sensors were analysed in the current mode, as depicted in Figure 4B. They collectively accounted for 99.99% of the total contribution, signifying a substantial role in the discrimination of peach fruit aromas in the experiment.

**Figure 5:**
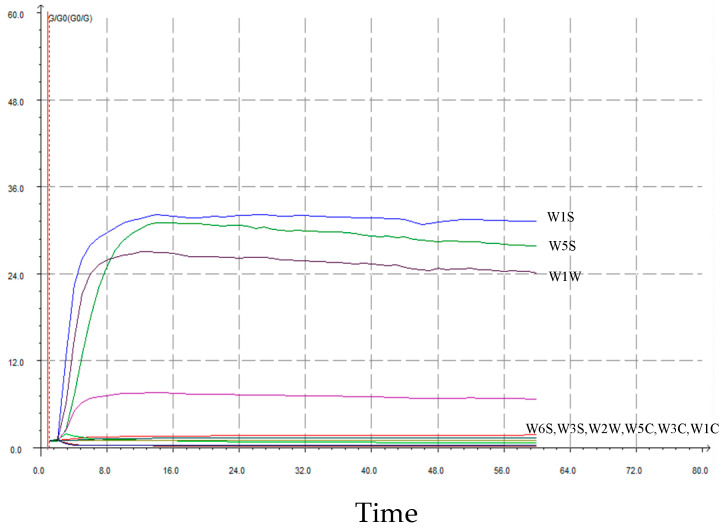
No.193 response values to different sensors of an electronic nose.

Figure 5 exhibits the response values of No.193 to the various sensors of an electronic nose, with peak values around at 24s. A stable period for further analysis was determined to be from 35 s to 37 s. Among the ten sensors, W1S (short-chain alkanes) exhibited the greatest response, followed by W5S (hydrocarbons and aromatic compounds), W1W (terpenes and organosulfur compounds), W2S (alcohols, ethers, aldehydes, and ketones), W6S (hydrogen), W2W (aromatic components and sulphur and chlorine compounds), W5C (nitrogen oxides), W3C (ammonia and aromatic compounds), and W1C (aromatic benzene). The largest response value was observed for W1S (32.702 ± 0.015), followed by W5S (32.803 ± 0.067), W1W (24.373 ± 0.102), and W2S (8.789 ± 0.005). The other sensors had lower response values of around 1. The coefficients of variation for W5C, W2W, and W1W were 1.24%, 0.74%, 0.39%, and 0.42%, respectively. W1C and W3S displayed relatively minor coefficients of variation, amounting to 0.14% and 0.04%, respectively. 

**Figure 6:**
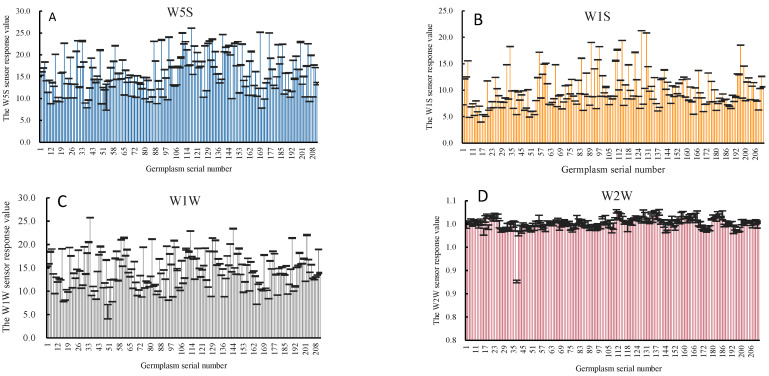

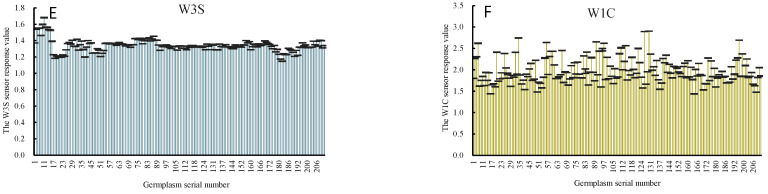
Main and aromatic sensors’ response values for Class I germplasms: (**A**), W5S sensitive to hydrocarbons, aromatic compounds; (**B**), W1S sensitive to short-chain alkanes such as methane; (**C**), W1W sensitive to terpenes and organosulfur compounds; (**D**), W2W sensitive to aromatic, sulfur and chlorine compounds; (**E**), W3S sensitive to long-chain alkanes; (**F**), W1C sensitive to aromatic benzene.

**Figure 7:**
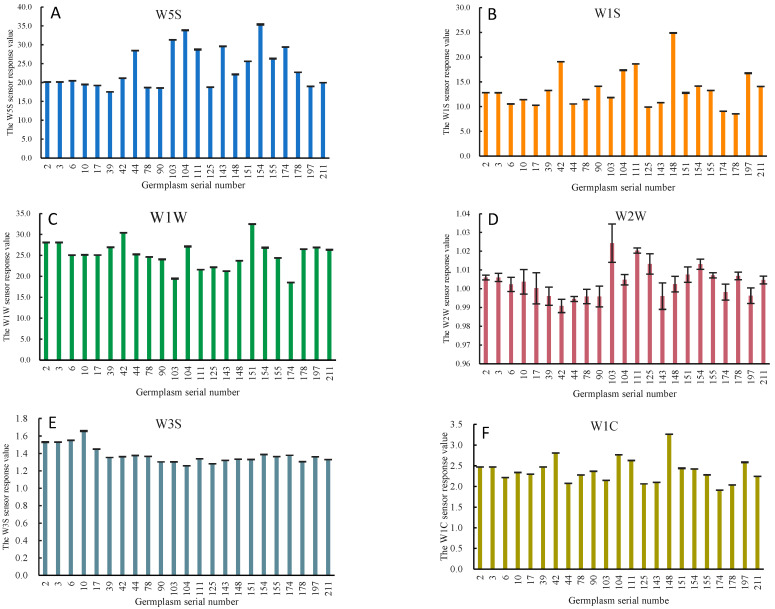
Main and aromatic sensors’ response values for Class II germplasms: (**A**), W5S sensitive to hydrocarbons, aromatic compounds; (**B**), W1S sensitive to short-chain alkanes such as methane; (**C**), W1W sensitive to terpenes and organosulfur compounds; (**D**), W2W sensitive to aromatic, sulfur and chlorine compounds; (**E**), W3S sensitive to long-chain alkanes; (**F**), W1C sensitive to aromatic benzene.

Figure 6 and Figure 7 show the stable signals for Classes I and II in the response values of the three primary sensors and the three aromatic sensors. The response values of the three main sensors differed significantly between Class I and Class II. Compared with the response values of the three aroma sensors, germplasms in Class I responded notably to the sensor W1C, while germplasms in Class II responded significantly to sensor W2W. This result was consistent with the previous results for the coefficient of variation. It indicates that the differences in peach aromas predominantly arose from non-aromatic substances such as methane, nitrogen oxides, and hydrogen sulphide, with variations in aromatic substances primarily reflected in the response values of W2W and W1C.

### 3.3. Sugars and Acids Identified via HPLC

**Figure 8:**
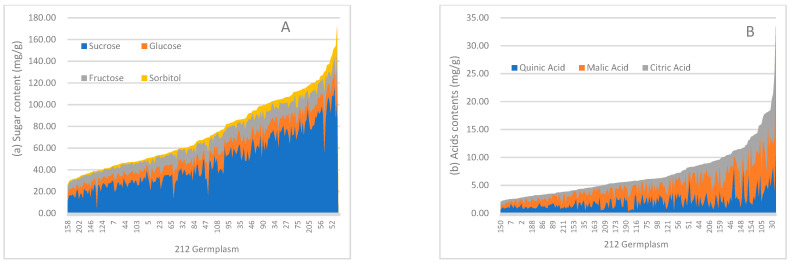
(**A**). Sucrose, glucose, fructose, and sorbitol content in 212 peach germplasms: blue area corresponds to sucrose scores; orange area corresponds to glucose; grey area corresponds to fructose; and yellow area corresponds to sorbitol. (**B**). Quinic, malic and citric acids content in 212 peach germplasms: blue area corresponds to quinic acid; orange area corresponds to malic acid; and grey area corresponds to citric acid.

Figure 8A presents the sucrose, glucose, fructose, and sorbitol content of 212 germplasms determined via HPLC, with sucrose ranging from constituting 11.9% to 83.3% (from 4.61 mg/g to 118.28 mg/g) of the total sugar content, while sorbitol corresponded to the lowest content, ranging from 0.01 to 26.80 mg/g (from 0% to 18.6%), in the 212 germplasms. The glucose and fructose percentages were similar, spanning from 3.8% to 47.7%. No.158 had the lowest total sugar content at 25.86 mg/g, whereas the highest content was 172.61 mg/g, corresponding to No.73. In Figure 8B, the total acid content varies from 2.07 mg/g in No.150 to 33.61 mg/g in No.76. The quinic acid content ranges from 0.32 to 10.72 mg/g, accounting for 5.6% to 76.3% of the total acid content. The malic acid content also showed similar ranges (0.40 to 19.26 mg/g) and percentages (from 12.9% to 79.6%) across the germplasms. No citric acid was detected in No.36, No.157, and No.203, while the highest citric acid content was found in No.64.

**Figure 9:**
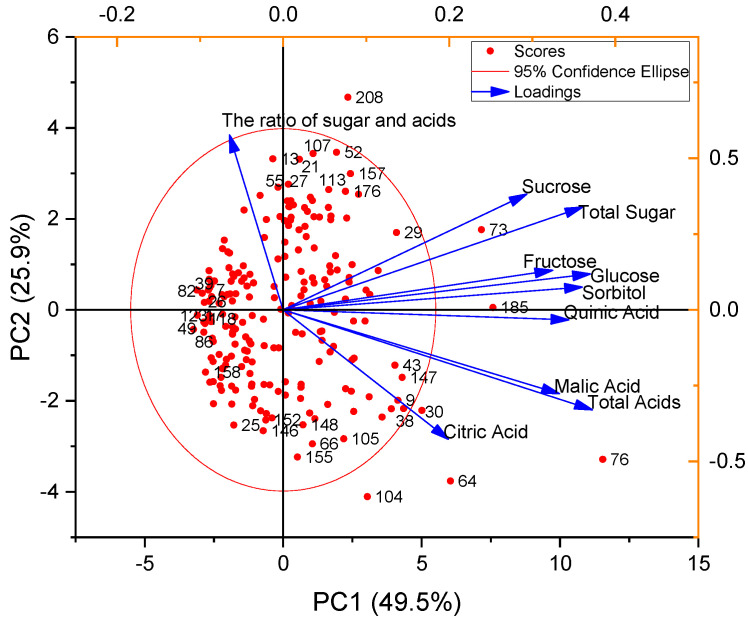
PCA of sugars and acids in 212 peach germplasm resources: red dot, 212 peach germplasms; blue arrow lines, loadings.

PCA was performed to assess the impact of sugars and acids on the evaluation of peach germplasms (Figure 9). The first principal component (PC1) accounted for 49.5% of the variance, with sugars and acids providing the largest contributions. The second principal component (PC2) contributed 25.9% to the variance, with the ratio of sugars to acids and sucrose contributing significantly. Quinic, malic, and citric acids had negative loadings for PC2. Thus, PC2 reflects a taste profile emphasizing sweetness. No.107 was characterized by sweetness with light sourness, but No.155 was sour with a hint of sweetness. No.49 had negative loadings for both PC1 and PC2. Additionally, a close relationship was found between sweetness and total sugars, similar to the relationship between malic acids and total acids. 

### 3.4. Comparison of Instrumental and Sensory Results

**Figure 10:**
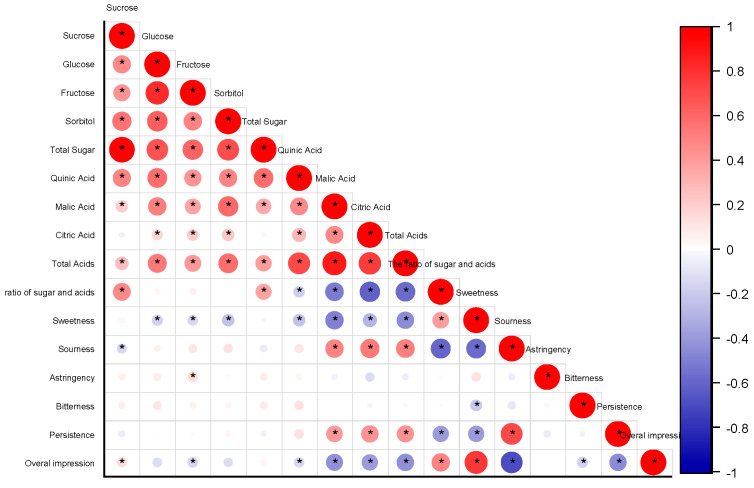
Correlation matrix heat map. Heat map of spearman correlation between sensory and HPLC analysis between each biomarker for the overall population analysed. * Indicated significant differences between instrumental and sensory parameters according to Pearson correlation coefficient (*p* < 0.05).

A negative correlation existed between sweetness and quinic, malic, and citric acids (r > −0.7), while a positive correlation was observed between sweetness and the ratio of sugar to acids (r < 0.6) (Figure 10). Sourness negatively correlated with sweetness and the ratio of sugar to acids but positively correlated with acids. Persistence showed a positive correlation with acids and sourness and a negative correlation with sweetness and the sugar-to-acid ratio. Overall impression correlated negatively with acids and sourness while correlating positively with sweetness and the sugar-to-acid ratio. 

## 4. Discussion

Fruit quality evaluation is an important component of peach fruit quality research. Sensory evaluation is commonly used to assess fruit quality. Based on the evaluation criteria, different samples are scored, allowing for the calculation of the mean values [20,21]. This study included the sensory determination of 212 peach germplasm resources, focusing on aroma, palate (sweetness, sourness, astringency, and bitterness), persistence, and overall impression. In 212 peach germplasm resources, the predominant taste profiles, ranked from highest to lowest, were sweetness, sourness, astringency, and bitterness (Figure 1A). No.134 exhibited the most pronounced sweetness, with an excellent impression, resulting from its high total sugar content and low total acids content. The long-finish germplasms (No.38 and No.47) presented high total acid content (over 15 mg/g) and a small ratio of sugar to acid (below 5). The PCA revealed the influence of each sensory descriptor on the evaluation of peach germplasm resources. PC1 reflected an enjoyable taste, while PC2 corresponded with negative feedback. The sensory evaluation highlighted that No.211 was sweet with a great impression, but No.101 was astringent and bitter, resulting in an unpleasant impression. The profile of No.119 included light sweetness, strong sourness, and a long finish, leading to a harsh impression. These findings reveal the importance of a strong characteristic aroma in shaping consumer preferences. However, some germplasms can present a faulty flavour because of an excessively strong aroma. An overly strong passion fruit flavour had the potential to present bromhidrosis in one sensory evaluation [22]. Thus, it was crucial to screen for peach germplasms with intense sweetness and an appropriate characteristic aroma to enhance peach quality.

An electronic nose (E-nose) is a simple, efficient, non-destructive, and stable device that imitates the human olfactory system. It has been used for assessing fruit quality and ripeness and in other applications [23,24]. In our research, W5S, W1S, and W1W could explain up to 99.99% of the total contributions, indicating that these non-aromatic substance classes (nitrogen oxides, methane, and hydrogen sulphide) distinguished the peach fruit aromas in our research. The distinction in aromatic substance classes corresponded mainly to the response values of two sensors (W2W and W1C); these values aligned with studies on pear and blueberry quality and aroma evaluation [25,26]. Upon comparing the results of the sensory evaluation and E-nose, the germplasms in Class I responded significantly to sensor W1C, which correlated with sensory perceptions of milk and caramel flavours. Germplasms in Class II showed a significant response to sensor W2W, associated with floral and herbaceous sensations. Notably, No.82, No.91, and No.193 were the most representative samples, with distinctive aromas of apple, butter/milk, and violet, respectively. These could serve as valuable indicators for fruit quality assessment and determining consumer preferences [27,28]. Thus, they could be used in developing new peach germplasm with excellent aroma profiles. 

Sucrose and malic acid were identified as the predominant components in peach germplasms, corroborating the results of previous studies [29]. The correlation between the sensory and HPLC results indicated that the fruits’ sweetness was primarily determined by their sugar content. The relative sweetness perceptions of fructose, sucrose, and glucose has been rated as 1.75, 1, and 0.75, respectively [16]. Despite high levels of sugars, peach sweetness was not necessarily pronounced because of sucrose. Sucrose made up the largest share of total sugars and did not contribute as strongly to sweetness. Additionally, the types of sugars and their ratio with respect to acids also affected perceived sweetness. Malic acid, which imparted a soft, refreshing, slightly irritating, and long-lasting sour–bitter taste, was the primary acid in peaches, while citric acid provided a pleasant, quickly dissipating sour taste. Three germplasms (No.36, No.157, and No.203) lacked citric acid, resulting in less sourness detected in their sensory evaluations. Furthermore, for the 212 peach germplasms studied, it was observed that a sugar-to-acid ratio exceeding 20 corresponded to sweetness scores above 5. This finding supports the results of the research by Colaric et al. [14], who suggested that a sugar-to-acid ratio ranging from 20 to 60 is typically associated with a moderate level of perceived sweetness. This ratio is important because it provides a balance of flavours, contributing to the overall sensory pleasure elicited by and the acceptability of peach fruit by consumers. By analysing the sugar-to-acid ratio, breeders and researchers can better understand and predict the sweetness profiles of peaches, constituting a crucial factor in consumer satisfaction and the marketability of the fruit.

## 5. Conclusions

In this study, we employed sensory evaluation, an electronic nose (E-nose), and high-performance liquid chromatography (HPLC) to assess fruit quality attributes such as aroma, sugars, and acids across 212 peach germplasm resources. These methods significantly contributed to the preliminary screening of peach germplasms with specific desirable characteristics. This study provides essential information that could facilitate the creation of superior germplasm resources. Therefore, evaluating and analysing the aroma and taste profiles of peach germplasms hold significant theoretical and practical value for the discovery, development, and utilization of high-quality peach resources. For future research, the integration of the electronic nose technique with headspace solid-phase microextraction (HS-SPME) and gas chromatography–mass spectrometry (GC-MS) could enhance the discrimination of peach fruit aroma components and aid in identifying the key aroma compounds within peach germplasm resources. This multidisciplinary approach may lead to a deeper understanding of the complex interactions between volatile compounds and contribute to the advancement of peach breeding programs targeting improved fruit aromas and flavour.

## Figures and Tables

**Table 1:** Electronic nose sensor array (PEN 3.5) using the portable E-nose (Air-sense Analytics GmbH, Germany).

Sensor Name	Sensor Sensitives
W1C	Sensitive to aromatic benzene
W3C	Sensitive to ammonia and aromatic compounds
W5C	Sensitive to nitrogen oxides
W1S	Sensitive to short-chain alkanes such as methane
W2S	Sensitive to alcohols, ethers, aldehydes, and ketones
W3S	Sensitive to long-chain alkanes
W5S	Sensitive to hydrocarbons and aromatic compounds
W6S	Sensitive to hydrogen
W1W	Sensitive to terpenes and organosulfur compounds
W2W	Sensitive to aromatic compounds and sulphur and chlorine compounds

**Table 2:** Special flavours in 212 peach germplasm resources.

Aroma Type	Flavour	Peach Germplasm Number
Fruity	Citrus	59, 181
	Apple	29, 54, 62, 91, 117, 163, 200
	Green Apple	18, 31, 208
	Honeydew	56, 161
	Mango	84, 99, 172, 182, 184, 196
	Apricot	207
	Juicy Honey Peach	188, 195, 205
	Yellow-Flesh Peach	147, 159, 167, 179, 197
	Passionfruit	8, 14, 19, 23, 100
	Lemon	15, 20, 87, 147
Floral	Gardenia	15, 30, 44, 45, 66, 185
	Jasmine	64, 178
	Rose	28, 63, 70, 99, 109, 144, 148, 183, 186
	Violet	22, 24, 41, 65, 68, 69, 71, 80, 83, 97, 106, 107, 108, 110, 112, 118, 120, 121, 122, 125, 127, 129, 130, 131, 132, 133, 134, 135, 137, 139, 141, 146, 171, 177, 189, 196, 197, 199, 202, 204, 210
Herbaceous or Vegetative	Herb	173
	Grass	34, 53, 92, 174, 176, 203, 206
	Celery	137
	Lotus Seed	168
	Carrot	118
Animal	Butter/Milk	9, 21, 55, 56, 59, 60, 61, 68, 76, 80, 86, 89, 91, 101, 112, 115, 128, 133, 134, 141, 144, 146, 152, 162, 165, 172, 176, 179, 181, 188, 196
Caramel	Candy	153, 189
	Popcorn	3, 57, 82, 94, 149
	Molasses	82, 201
	Honey	58, 165
Nutty	Peanut	32, 74, 78, 114, 143, 154
	Cereal	37, 145
	Red Bean	13
Woody	Smoky	15, 139, 152
	Rubber	111
Pungent	Metal	50, 71, 190, 195
Faults	Spoiled	38, 50, 76
	Off-Odour	11, 123, 160, 199

## Data Availability

Data are contained within the article.

## Appendix A. Germplasm Resource Information of 212 Peaches

**Number**	**Germplasm Name**	**Country of Origin**	**Aroma**	**Palate and Taste**				**Persistence**	**Overall Rating**	**The Ratio of Sugars to Acids**
				**Sweetness**	**Sourness**	**Astringency**	**Bitterness**			
1	Li Xia Hong	CHN	3.5	5	2	0	0	4	5	20.3
2	Chun Lei	CHN	4	3.9	0	0	2.4	3.2	4	21.3
3	Zao Shuo Mi	CHN	4.4	5.8	2.3	0	0	4.7	5.3	18.8
4	Zao Mei	CHN	3.8	4.6	1.8	1.3	0	4.5	5.1	22.8
5	Chun Hua	CHN	3.6	3.8	2.4	0	0	3.6	4.2	15.5
6	Hu 021	CHN	4	4.1	2.5	0	0	2	2.8	17.8
7	Zao Xia Lu	CHN	3	4.4	3	0	0	4	4.6	17.2
8	04-13-40	CHN	5.5	5.2	2.6	0	0	4	5.2	14.8
9	Yun Long Shui Mi	CHN	7.5	8	0	0	0	5	6.3	5.8
10	Zao Lu Pan Tao	CHN	5.1	6	0	2.1	0	5.3	5	20.5
11	Jing Chun	CHN	7.5	4.8	3	2.5	2	4.8	2	16.0
12	Chun Yan	CHN	6	5	0	2	1.8	4.9	4	12.6
13	82-18-73	CHN	6.2	7.8	0	0	0	5	6	32.6
14	Mayfire	USA	7	3	6	0	0	6	2.3	8.3
15	SpringCrest	USA	4.8	5	4	0	0	6.1	5	7.6
16	Jin Shan Zao Hong	CHN	7	6.8	0	0	0	4	4.8	14.3
17	Takeiwasehakuho	JPN	6.2	5.2	0	0	0	4.3	4.5	14.4
18	Zao Hong Zhu	CHN	6.3	6.4	2	0	0	3.8	4	17.9
19	Hua Guang	CHN	2	3	1.8	0	1.8	4	2	13.2
20	Shu Guang	CHN	3	6.2	3	0	0	5	4.8	10.6
21	Saotome	JPN	6	7.5	0	0	0	3	5.4	27.7
22	Springtime	USA	3	5	7	0	0	6	2.8	4.6
23	Bai Xiang Lu	CHN	6.5	6.5	0	0	0	3	3.4	15.3
24	Jin Shan Zao Lu	CHN	7	7.4	0	0	0	3	3.8	8.0
25	Armking	USA	2.5	4	5.8	0	0	4.8	5	3.6
26	Xia Hui 3	CHN	7.4	7.4	0	1.8	0	3.2	4.1	15.9
27	Sha Ji 2	CHN	4.2	6.2	0	0	0	2.8	6	26.0
28	Zao Hong Bao Shi	CHN	3.8	7.6	0	0	0	3	6.3	12.3
29	Tsukuba 86	JPN	2.4	4.8	1.2	1	0	3.1	5.2	13.6
30	Flordacrest	USA	6.4	4.9	6.2	0	1.3	5.8	4.4	5.5
31	Sunsplash	USA	0	3.2	6	0	0	5.8	4.1	6.1
32	NJN72	USA	6.8	6.8	3.3	0	0	4.9	4.1	6.4
33	Robin	USA	7.1	7.6	0	0	0	3	6.1	21.5
34	Favolate 3	ITA	7.4	5	3.2	0	0	4.1	4.2	10.0
35	Hui Yu Lu	CHN	7.5	7.1	0	0	0	3	6.5	19.7
36	Yang Zhou Zao Tian Tao	CHN	3.2	4.1	1.8	0	0	2.1	3.8	15.1
37	Kun Ming Hong Rou Tao	CHN	0	2.8	0	0	3.1	3	2.1	16.9
38	Mang Zhong Lu	CHN	0	1.9	7.2	0	0	8	1.1	5.0
39	Xia Hui 1	CHN	3.2	6.3	1.8	1.1	0	2.6	4.8	17.9
40	Spring Snow	USA	4.1	4.5	2.6	0	0	4.5	3.3	13.7
41	Tropic Prince	USA	6.1	3.8	6	0	0	6	5.2	8.3
42	Flordaking	USA	7.2	7.1	4.9	0	0	4.8	4.5	5.5
43	Crimsonbaby	USA	4.8	3.4	6.9	0	0	5.1	2.5	7.2
44	TX2B7N	USA	5	7.2	7.1	0	0	6.2	3.2	5.4
45	Zi Jin Hong 1	CHN	7.1	5.9	2.6	0	1.1	2.1	4.8	10.5
46	Babiole	FRA	6.9	4.2	6.3	0	0	5.8	2.3	8.8
47	Hong Tao	CHN	0	1.8	7.2	0	0	8.2	1	4.4
48	85-13-24	CHN	9.5	7.5	1.9	0	0	2.1	3.4	8.4
49	63-15-75	CHN	1.2	6.8	0	1.2	0	1.8	4.2	12.8
50	Hong Rou Tao 1	CHN	0	0	6.1	0	0	5.8	0.9	13.0
51	Li Jiang Tao	CHN	0	6.8	0	1.8	0	1.6	4.5	13.7
52	TX4C199	USA	0	7.4	0	0	0	2.1	6.8	26.4
53	Yu Hua 3	CHN	4.6	8	0	0	0	1.1	5.4	13.0
54	Jing Ling Huang Lu	CHN	4.8	6.4	3	0	0	3	5.2	17.9
55	An Nong Shui Mi	CHN	6.2	7.4	0	1.2	0	1.4	5.8	25.5
56	Jiang Jin IV2-9	CHN	6.5	6.9	0	1.1	0	1.2	5.6	17.5
57	Hikawa Hakuhou	JPN	7.1	7.7	0	0	0	4.8	6.6	19.3
58	Early Chinese Cling	CHN	3.8	5	6.1	3	0	2.8	2	11.8
59	Hatsukami	JPN	6.6	3.9	6.2	3.6	0	5.8	1.2	7.5
60	Ying Hua Lu	CHN	6.8	7.2	0.5	1.2	0	2.1	5.6	19.9
61	Early White Giant	USA	5.8	4.8	6.3	1	0	3	2.3	12.9
62	TX4C189LN	USA	0	6.4	3.1	0	0	3	6.5	28.8
63	Mei Gui Hong	CHN	2.5	6.8	2	0	0	2.1	6	10.8
64	NJN70	USA	2	2	5.2	0	0	5	1.2	4.5
65	Hu You 018	CHN	4.8	6.9	0	0	0	1.8	5	19.9
66	Red June	USA	6	4.9	6.2	0	0	5.3	1.4	4.1
67	Hu You 003	CHN	3.4	7.4	2.1	0	0	2.1	6.1	18.7
68	Rui Guang 22	CHN	6.9	5.2	3.2	0	0	1.8	3.8	9.0
69	Quetta	IND	3.8	1.8	7.3	0	0	6.6	1.2	5.9
70	TX4C188LWN	USA	6.6	6.9	1.1	0	0.6	1.4	6.3	8.4
71	TX2C104N	USA	5	2.1	7	0	0	7.1	0.6	5.6
72	Sunraycer	USA	4.2	4.2	5.8	0	0	4.5	1.1	6.4
73	Xian Yang Hong Rou Tao	CHN	2.1	4.8	3.9	0	0	2.1	1.8	12.0
74	Xiao Hong Pao	CHN	1.2	5.4	0	0	0	0.8	3.1	21.7
75	Shen Nong Hong Rou	CHN	0	3	2.1	0	1	2.8	1.6	18.4
76	Bei Jing Yi Xian Hong	CHN	1.6	0	6	0	0	5.8	0.1	3.9
77	Ye Ji Hong	CHN	3.8	4.4	0	0	0	2.1	2.1	10.4
78	Tang Shan Tao	CHN	0	4.8	0	0	1.2	1.1	3.4	9.2
79	Jie Tao	CHN	0	6.3	0	0	0	0.8	5.1	6.5
80	Jin Xiang	CHN	4.6	7	0	0	0	3	6.8	15.6
81	Chiyohime	JPN	2.1	4.8	0	0	0	1.2	5.9	21.3
82	Nunomewase	JPN	3	5	0	0	0	0.8	4.4	18.9
83	Zao Jin Lu	CHN	2	4.9	0	0	0	5.1	3.8	15.8
84	Jin Hua Lu	CHN	1.8	6.8	0	0	0	6.2	7.1	24.7
85	Jiu Yan	CHN	0	7.2	0	3.2	0	1.4	6.4	7.5
86	Jing Hong	CHN	4.2	7.1	0	1.3	0	3	6.8	11.6
87	Bao Pi Yang Tao	JPN	6.8	1.8	7.2	0	0	5.6	0.2	6.5
88	Bei Nong 2	CHN	7.2	4.8	7.2	0	0	3.2	1.2	18.05
89	Natsuki	JPN	3.8	4.8	1	0	0	2.8	1.4	23.25
90	Fertini Morettini	ITA	3.2	4.9	7.2	1.2	0	6	2.1	10.5
91	Zhe Jin 2	CHN	7	4.5	7.2	0	0	6	1.1	7.2
92	Spring Gold	USA	5	4.9	6	0	0	5.8	3.2	5.1
93	85-13-19	CHN	4.4	7.1	3	0	0	3.2	5.8	6.3
94	Jin Xu	CHN	4.4	4.9	5	4.2	0	3.3	2.2	11.6
95	Nagin 1	JPN	0	5	4.8	0	0	4.9	3.4	23.2
96	Yu Hua Lu	CHN	2.1	4.8	0	0	0	3	2.1	8.9
97	Yuan Dong Bai Tao	CHN	1.4	7.4	0	0	0	3	7.6	14.4
98	Tou Xin Hong	CHN	0	7.4	5	0	0	3	4.3	13.9
99	Sunking	USA	6	5.2	7.1	0	0	5.2	1.1	5.9
100	NJN76	USA	7.4	5	6.3	0	0	5.2	3.8	9.4
101	Liu Yang Si Jin Tao	CHN	1.8	6.3	2.2	3.2	0.9	3.1	3.2	10.5
102	Pegaso	ITA	4.8	4.2	7.4	0	0	6	2.8	5.1
103	Sunblaze	USA	2.1	0	7.2	0	0	3.5	0.2	5.1
104	Flordaglo	USA	7	4.8	7.4	0	0	6.8	4.3	2.8
105	Maravilha	USA	6.9	5	6.8	0	0	5.4	0.3	5.1
106	Hu You 004	CHN	0	7.1	2	0	0	4.2	6.8	19.6
107	Hu You 007	CHN	3.2	5.1	0	0	0	2.3	5.9	28.3
108	Zi Jing Hong 2	CHN	1.8	6.8	2.1	0	0	5.4	6.1	27.2
109	Early Red 2	USA	6.8	6.7	5.8	0	0	5.8	4.8	7.3
110	Sheng Guang	CHN	2.4	6.4	1.8	2.1	0	2	6.6	10.3
111	801	CHN	7.2	7.4	2.6	0	0	4.3	7.1	16.8
112	Rui Pan 1	CHN	6.8	7.4	0	0	0	3	6.8	18.5
113	Zao Feng Wang	CHN	0	6.8	0	0	0	2.8	6.1	21.5
114	Kana Iwa	JPN	0	7.4	0	0	0	2.4	6.9	16.0
115	Da Zhao Huang Tao	CHN	5.8	7.4	0	0	0	2.3	8.3	21.6
116	Mei Ting	CHN	2.1	4.8	0	0	0.9	2.8	4.5	17.4
117	Qu Jing Tian Tao	CHN	1.8	4.6	3.1	0	0	2.8	2.2	6.1
118	63-15-59	CHN	1.3	7.3	0	0	0	5.4	8.1	13.0
119	Yi Xian Bai	CHN	0	0	7.4	0	0	8.9	0.2	4.8
120	Rui Guang 7	CHN	1.2	4.2	5.4	0	0	3.1	2.1	11.9
121	Ao19	AUS	4.3	5	6.1	0	0	5.4	1.9	7.3
122	Rui Guang 19	CHN	6.9	7.2	3.1	0	0	3	6	18.0
123	Rui Guang 2	CHN	4.3	6.1	0	0	0	2.1	7.6	13.0
124	Feng Huang	CHN	7	6.5	0	0	0	6.2	7.8	7.6
125	Redhaven	USA	6.8	5	6.8	0	0	5.1	4.2	11.9
126	Vesuvio	USA	4.2	4.8	4.5	1.1	0	3.2	3.2	10.6
127	Sunfre	USA	0	1.8	6.2	0	0	5.4	0.8	5.5
128	Silver Load	USA	4.1	4.3	3.8	0	1.2	3.4	2.8	8.2
129	Galaxy	USA	4.2	6.5	0.8	0	0	3	7.7	13.2
130	TX4D170	USA	1.8	1.8	7	0	0	6	0.8	6.7
131	Ban Han	CHN	7	2	0	0	0	1.1	1.3	12.2
132	Xia Hui 5	CHN	5.8	6.8	0	0	0	1.4	8.2	22.9
133	Mei Guo Hong Pan Tao	USA	6.1	7.4	0	0.8	0	2.3	6.8	14.7
134	UFO4	ITA	9.2	9.2	0	0	0	6.3	9.4	18.2
135	Saturn	USA	1.3	6.8	0	0	0	2.1	6.7	11.3
136	Jin Jiu Hong	CHN	4.3	6.1	3	4.2	0	3.2	3.2	17.9
137	Nectaross	USA	0	3.1	0	0	1.8	0.3	1.1	7.5
138	Hong Gan Lu	CHN	3.2	6.9	0	0	0	1.2	6.1	23.2
139	You Dao	CHN	6.8	6.5	3	0	0	2.5	6.3	21.5
140	Rui Guang 2	CHN	4.3	4.8	2.3	0	0	2	5.8	6.1
141	Yue 172	CHN	4.3	7.2	0	0	0	1.1	7.3	6.3
142	Qing Pi Qiu Tao	CHN	4.4	5.3	0	0	0	1.2	4.8	23.3
143	Qing Guang	CHN	3.1	7.4	3	0	0	3	6.9	13.8
144	Crimson Gold	USA	0	4.3	6.2	0	0	5.8	2.3	5.2
145	Rui Guang Mei Yu	CHN	1.2	4.1	1.8	0	0	3.4	3.1	10.4
146	Fuzalode	FRA	6.9	3.2	7.2	0	0.9	5.6	0.6	3.9
147	TX4F244C	USA	3.2	4.8	7.2	0	0	6	2.1	7.6
148	Rose Princess	USA	1.3	5	5.3	0	0	5.1	1.2	5.0
149	Da Hong Pao	CHN	7.3	6.9	0	1.3	0	5.3	6.1	5.3
150	Da Tian Tao	CHN	0	5	1.8	0	0	3	3.1	19.2
151	Yi Xian Hong	CHN	1.1	4.3	0	0	1	2.1	2.4	12.4
152	Lu Shui Tao 6	CHN	1.2	4.3	1.3	0	0	3.2	4.2	4.8
153	Xai Cui	CHN	0	6.9	0	0	0	5	5.8	24.3
154	Tian Jin Shui Mi	CHN	0	0	5.1	0	0	2.8	1.2	5.6
155	Flavor Gold	USA	4.8	4.9	6.2	0	0	4.8	0.8	3.7
156	Xiang Jin Pan	CHN	2.1	4.8	5.5	0	0	5.8	3.3	16.6
157	Xia Hui 6	CHN	1.8	5.5	0.8	0	0	1.5	6.2	21.2
158	Lu Lin	CHN	0	4.8	0	0	0	1.1	4.2	6.9
159	Huang Nian He	CHN	4.1	5.5	1.8	0	1.8	2.4	3.1	11.4
160	Miyako Hakuhou	JPN	3.8	5.2	0	0	0	2.1	4.3	19.5
161	Tsukuba 89	JPN	1..2	5	1.2	1.8	0	2.1	2.6	19.1
162	Asama Hakuto	JPN	4.6	7.1	0	0	0	1.4	7.6	10.1
163	Xiao Hong Hua	CHN	1.3	6.5	1.1	0	0	2.1	6.8	21.9
164	Matsumori	JPN	1.5	3.4	1.4	0	0	0	3.1	8.9
165	Ta Qiao	CHN	5.8	8.6	2.5	0	0	3.5	5.8	18.6
166	Cullinan	USA	0	2.1	5	2.2	1.2	5.8	2.2	10.7
167	Harken	CAN	7.1	5.6	4.8	1.8	0	5.6	1.9	12.2
168	Frederica	USA	5.2	3.8	6.2	0	0	6.3	0.8	12.8
169	Vista Rich	ESP	0	2.4	6.9	0	0	6.2	2.2	5.9
170	Redtop	USA	0	4.6	7.5	0	0	6.8	1.8	9.4
171	Lisbeth	HUN	1.8	1.8	7.4	0	0	6.5	1.4	9.1
172	Catherina	ESP	7.2	6.2	1.5	0	0	4.2	5.4	9.4
173	Zao Shu Huang Gan Tao	CHN	6.5	1.8	4.8	0	1.1	3.8	1.8	8.3
174	Blaze Prince	USA	0	5.4	4.8	0	0	4.1	2.1	6.0
175	Rumiana	BGR	5.1	6.1	4.8	0	0	3.2	1.1	4.9
176	Rui Pan 5	CHN	3.8	7.6	0	2.1	0	2.1	7.4	19.6
177	Rui Pan 3	CHN	2.1	6.8	0	0	0	1.8	6.5	14.6
178	Yu Xia Pan Tao	CHN	1.8	5.2	0	0	0	1.2	5.5	15.3
179	Jin Xia Pan Tao	CHN	7.2	7.4	0	0	0	1.5	6.8	16.5
180	63-13-35	CHN	0	4.5	0	1.2	0	1.8	5.2	15.9
181	Babygold 5	USA	6.8	5.2	4.3	0	0	3	3.2	6.5
182	Babygold 6	USA	7.2	7.2	5	0	0	3.8	3.1	11.7
183	Stark Delicious	USA	3.2	3.2	6.3	0	0	4.3	2.2	9.3
184	NJC3	USA	6.1	6.1	7.2	0	0	3.5	4.5	6.3
185	Fa Guo You Tao	FRA	4.8	1.8	6.4	0	0	4.3	1.2	8.5
186	Stark Sunglo	HUN	5.1	2.1	6.5	0	0	3.8	1.8	5.1
187	Yoshihime	JPN	1.8	4.9	3.8	2.1	0	3.4	4.1	19.0
188	Xia Hui 7	CHN	6.4	5.8	0	0	0	1.1	6.2	17.3
189	Yang Shan 2	CHN	5	7.5	0	0.9	0	1.3	7.6	22.3
190	Jing Feng	CHN	7.2	6.8	2.5	1.4	0	3	5.4	10.8
191	Gan Xuan 4	CHN	0	2	0	0	0	1.4	1.8	15.8
192	Kanto 5	JPN	6.9	6.8	0	0	0	1.2	6.2	13.2
193	Xi Zhuang 1	CHN	7.2	5	4.2	0	0	3.5	4.6	11.1
194	Yellow St.John	USA	5.1	5	7.5	0	0	6.8	2.1	8.0
195	Yu Hua 2	CHN	7	7.6	0	3.2	0	2.4	6.8	11.0
196	Shasta	USA	8.2	6.2	7.3	0	0	6.2	1.8	5.3
197	McNeely	USA	6.8	5.2	6.9	0	0	3.5	1.6	5.8
198	Harbrite	CAN	5	4.8	6.8	0	0	5.8	2.1	6.6
199	Da Jie Tao	CHN	8.9	6.4	0	0.9	0	1.4	5.8	13.9
200	Hei You Tao	CHN	0	5.8	6.1	4.3	0	5.4	3.2	6.9
201	Dixon	USA	4.8	6.8	1.2	3.3	0	3	4.8	10.5
202	Veteran	CAN	6.3	6.9	0	0	0	1.1	6.8	5.8
203	Nan Shan Tian Tao	CHN	1.8	6.8	1.2	0	0	1.6	4.8	15.0
204	Huang Rou Pan Tao	CHN	4.3	7.2	0	2.2	0	1.1	5.8	16.5
205	Xi Shan Pan Tao	CHN	3.5	6.7	0	1.2	0	0.8	6.5	18.8
206	Qiu Kui	CHN	0	4.2	6.2	1	0	4.5	3.2	6.4
207	Compact Roman	USA	2.2	3.8	5.4	0	0	3.8	2.8	11.9
208	Xia Hui 8	CHN	0	6.4	0	0	0	1.2	8.8	36.4
209	Jin Xiu	CHN	2.5	7.4	0	0	0	2.4	9.2	13.3
210	Shen Zhou Hong Mi	CHN	3.1	4.8	6.4	0	0	3	3.8	8.6
211	A Chu Tao	JPN	0	8.6	0	0	0	1.8	8.8	8.2
212	Mika	JPN	0	7.3	0	1.4	0	1.2	6.8	11.8
		Note: CHN, China; USA, the United States of America; JPN, Japan; CAN, Canada; FRA, France; ESP, Spain; ITA, Italy; BGR, Bulgaria; IND, India; AUS, Australia; HUN, Hungary.								
